# An Assessment of Ovarian Cancer Histotypes Across the African Diaspora

**DOI:** 10.3389/fonc.2021.732443

**Published:** 2021-11-26

**Authors:** Sophia H. L. George, Ayodele Omotoso, Andre Pinto, Aisha Mustapha, Alex P. Sanchez-Covarrubias, Usman Aliyu Umar, Ali Bala Umar, Timothy Abiola Oluwasola, Clement Abu Okolo, Umeh Uchenna Anthony, Francis Ikechukwu Ukekwe, Maisaratu A. Bakari, Aminu M. C. Dahiru, Habiba Ibrahim Abdullahi, Bawa Ahmed Abimiku, Aisha Abdurrahman, Asmau Usman, Saad Aliyu Ahmed, Hadiza Abdullahi Usman, Abba Kabir, George Uchenna Eleje, Michael Emeka Chiemeka, Emily Nzeribe, Ikechukwu Nweke, SaiduAbubakar Kadas, Dauda E. Suleiman, Etim Ekanem, Umemmuo Maureen Uche, Jibrin Paul, Uzoma Maryrose Agwu, Felix O. Edegbe, Rose I. Anorlu, Adekunbiola Banjo, Kayode Olusegun Ajenifuja, Adegboyega Adisa Fawole, Ibrahim O. O. Kazeem, Francis Magaji, Olugbenga Silas, Boma Precious Athanasius, Nyengidiki Kennedy Tamunomie, Emem Bassey, Kunle Abudu, Ibrahim G. Ango, Kabiru Abdullahi, Ishak Lawal, Suleiman Aliyu Kabir, Victor Ekanem, Michael Ezeanochie, Usman Rahman Yahaya, Melissa Nicole Castillo, Vishal Bahall, Vikash Chatrani, Ian Brambury, Saida Bowe, Darron Halliday, George Bruney, Raleigh Butler, Camille Ragin, Folakemi Odedina, Srikar Chamala, Matthew Schlumbrecht, Bala Audu

**Affiliations:** ^1^ Department of Obstetrics, Gynecology and Reproductive Sciences, Division of Gynecologic Oncology, Sylvester Comprehensive Cancer Center, University of Miami Miller School of Medicine, Miami, FL, United States; ^2^ African Caribbean Cancer Consortium, Philadelphia, PA, United States; ^3^ Transatlantic Gynecologic Cancer Research Consortium, Bauchi, Nigeria; ^4^ Department of Pathology, University of Calabar Teaching Hospital, Calabar, Nigeria; ^5^ Department of Pathology, University of Miami Miller School of Medicine, Miami, FL, United States; ^6^ Department of Obstetrics and Gynecology, Gynecologic Oncology Unit, Ahmadu Bello University Teaching Hospital, Zaria, Nigeria; ^7^ Department of Pathology, Aminu Kano Teaching Hospital, Kano, Nigeria; ^8^ Department of Obstetrics and Gynecology, Gynecological Oncology Unit, College of Medicine, University of Ibadan, Ibadan, Nigeria; ^9^ Department of Pathology, College of Medicine, University of Ibadan, Ibadan, Nigeria; ^10^ Federal Medical Centre Katsina, Katsina, Nigeria; ^11^ Department of Obstetrics and Gynecology and Department of Pathology, University of Nigeria Teaching Hospital Enugu, Enugu, Nigeria; ^12^ Department of Obstetrics and Gynecology and Department of Pathology, Federal Medical Center Yola, Yola, Nigeria; ^13^ Department of Obstetrics and Gynecology and Department of Pathology, University of Abuja Teaching Hospital, Gwagwalada, Nigeria; ^14^ Department of Pathology, Ahmadu Bello University Teaching Hospital, Zaria, Nigeria; ^15^ Department of Obstetrics and Gynecology, University of Maiduguri Teaching Hospital, Maiduguri, Nigeria; ^16^ Department of Obstetrics and Gynecology, Nnamdi Azikiwe University Teaching Hospital, Nnewi, Nigeria; ^17^ Department of Anatomic Pathology and Forensic Medicine, Nnamdi Azikiwe University Teaching Hospital, Nnewi, Nigeria; ^18^ Department of Obstetrics and Gynecology and Department of Pathology, Federal Medical Center, Owerri, Nigeria; ^19^ Department of Obstetrics and Gynecology and Department of Pathology, Abubakar Tafawa Balewa University Teaching Hospital, Bauchi, Nigeria; ^20^ Department of Obstetrics and Gynecology and Department of Pathology, National Hospital, Abuja, Nigeria; ^21^ Department of Obstetrics and Gynecology and Department of Pathology, Alex Ekwueme Federal University Teaching Hospital Abakaliki, Abakaliki, Nigeria; ^22^ Department of Obstetrics and Gynecology and Department of Pathology, Lagos University Teaching Hospital, Lagos, Nigeria; ^23^ Department of Obstetrics and Gynecology and Department of Pathology, Obafemi Awolowo University Teaching Hospital, Ile-Ife, Nigeria; ^24^ Department of Obstetrics and Gynecology and Department of Pathology, niversity of Ilorin Teaching Hospital, Ilorin, Nigeria; ^25^ Department of Obstetrics and Gynecology and Department of Pathology, Jos University Teaching Hospital, Jos, Nigeria; ^26^ Department of Anatomical Pathology, University of Port Harcourt Teaching Hospital, Port Harcourt, Nigeria; ^27^ Department of Obstetrics and Gynaecology, University of Port Harcourt Teaching Hospital, Port Harcourt, Nigeria; ^28^ Department of Obstetrics and Gynecology and Department of Pathology, University of Uyo Teaching Hospital, Uyo, Nigeria; ^29^ Department of Obstetrics and Gynecology and Department of Pathology, smanu Danfodiyo University Teaching Hospital Sokoto, Sokoto, Nigeria; ^30^ Department of Obstetrics and Gynecology and Department of Pathology, Federal Medical Center - Birnin Kebbi, Birnin Kebbi, Nigeria; ^31^ Department of Anatomic Pathology, University of Benin Teaching Hospital, Benin City, Nigeria; ^32^ Department of Obstetrics and Gynecology, University of Benin Teaching Hospital, Benin City, Nigeria; ^33^ Department of Obstetrics and Gynecology, Federal Teaching Hospital Gombe, Gombe, Nigeria; ^34^ Department of Gynecologic Oncology, University of West Indies, Port-of-Spain, Trinidad and Tobago; ^35^ Faculty of Medical Sciences, Department of Gynecologic Oncology, University of West Indies-Cave Hill, Bridgetown, Barbados; ^36^ Department of Obstetrics and Gynecology, University of West Indies-Mona, Kingston, Jamaica; ^37^ Princess Margaret Hospital, University of the West Indies, School of Clinical Medicine and Research, Nassau, Bahamas; ^38^ Cancer Prevention and Control Program, Fox Chase Cancer Center, Philadelphia, PA, United States; ^39^ Department of Pharmacotherapy and Translational Research, University of Florida, Orlando, FL, United States; ^40^ Department of Pathology, Immunology and Laboratory Medicine, University of Florida, Gainesville, FL, United States

**Keywords:** ovarian cancer, black women, germ cell, Caribbean, Nigeria, sex cord stromal, epithelial ovarian cancer (EOC)

## Abstract

**Objective:**

Ovarian cancer in Black women is common in many West African countries but is relatively rare in North America. Black women have worse survival outcomes when compared to White women. Ovarian cancer histotype, diagnosis, and age at presentation are known prognostic factors for outcome. We sought to conduct a preliminary comparative assessment of these factors across the African diaspora.

**Methods:**

Patients diagnosed with ovarian cancer (all histologies) between June 2016-December 2019 in Departments of Pathology at 25 participating sites in Nigeria were identified. Comparative population-based data, inclusive of Caribbean-born Blacks (CBB) and US-born Blacks (USB), were additionally captured from the International Agency for Research on Cancer and Florida Cancer Data Systems. Histology, country of birth, and age at diagnosis data were collected and evaluated across the three subgroups: USB, CBB and Nigerians. Statistical analyses were done using chi-square and student’s t-test with significance set at p<0.05.

**Results:**

Nigerians had the highest proportion of germ cell tumor (GCT, 11.5%) and sex-cord stromal (SCST, 16.2%) ovarian cancers relative to CBB and USB (p=0.001). CBB (79.4%) and USB (77.3%) women were diagnosed with a larger proportion of serous ovarian cancer than Nigerians (60.4%) (p<0.0001). Nigerians were diagnosed with epithelial ovarian cancers at the youngest age (51.7± 12.8 years) relative to USB (58.9 ± 15.0) and CBB (59.0± 13.0,p<0.001). Black women [CBB (25.2 ± 15.0), Nigerians (29.5 ± 15.1), and USB (33.9 ± 17.9)] were diagnosed with GCT younger than White women (35.4 ± 20.5, p=0.011). Black women [Nigerians (47.5 ± 15.9), USB (50.9 ± 18.3) and CBB (50.9 ± 18.3)] were also diagnosed with SCST younger than White women (55.6 ± 16.5, p<0.01).

**Conclusion:**

There is significant variation in age of diagnosis and distribution of ovarian cancer histotype/diagnosis across the African diaspora. The etiology of these findings requires further investigation.

## Introduction

Globally, ovarian cancer remains a deadly disease (1). In 2021, GLOBOCAN estimates there will be 313,959 new diagnoses of ovarian cancer worldwide (2, 3). Ovarian cancer is a heterogeneous disease with three major histologic types: epithelial ovarian cancer (EOC), germ cell tumors (GCT), and sex cord stromal tumors (SCST). EOC is the most diagnosed histologic type and high-grade serous ovarian carcinoma (HGSC) is the most common epithelial tumor. EOCs like HGSC are aggressive and are usually diagnosed at advanced stages with poor overall survival. GCTs and SCSTs are rare, diagnosed at early stages and have relatively better overall survival. Women of African ancestry have a low incidence of ovarian cancer (1, 4–6). Unfortunately, like many other cancer diagnoses, Women of West African ancestry (Black women) with ovarian cancer experience worse outcomes than White women. In the US, Black women have higher morbidity and mortality rates and higher un-staged or unclassified tumors compared with White women, resulting in undertreatment with subsequent compromise in progression-free survival (5, 7, 8). The 5-year ovarian cancer survival rate is 51% for Black women under 65 years of age and 22% for those 65 years and older. In contrast, among White women, these rates go up to 60% and 29% respectively (5, 9). Between 2005 and 2014, the age-adjusted incidence of ovarian cancer decreased by 1.4% in non-Hispanic Whites, but was stable in Blacks, Hispanics, American Indians and other ethnic groups (10). In addition to differential outcomes, recent data suggest that there are differences in the proportion of ovarian cancer histologies diagnosed in Black women in general (8, 11).

The Transatlantic slave trade was the largest forced immigration in history, transporting Africans to the US and the Caribbean (10). In the US, non‐Hispanic Blacks comprise the second‐largest racial/ethnic minority group and are disproportionately affected by high cancer mortality and morbidity. The Black population in the US is polylithic and is composed of both US native-born Black (UBB) and immigrant Black populations from the Caribbean (CBB) and Africa. Blacks from the US and the Caribbean have predominant West African ancestry, the majority from Nigeria, Ghana, and Benin (12). Understanding the global distribution of ovarian cancer histologic types among women of African ancestry is important to identify modifiers of disease etiology as well as opportunities for therapeutic interventions; both can improve ovarian cancer outcomes in a number of different treatment environments.

Notwithstanding the social, economic and health inequities linked to ovarian cancer outcomes in women of African descent in the US, we proceeded to study the prevalence of ovarian cancer histologies and age at diagnosis across the African diaspora. In this pilot study, our two-fold objective is first, to evaluate population-level patterns of ovarian cancer in Black women both globally and locally, and second, to describe the correlative patterns between age and histologic distribution in our TAGCReC-member cohorts. Such data may suggest variable etiologies of disease and generate hypotheses regarding global biologic variations in ovarian cancer in Black women.

## Methods

### Ethical Approval

This study was approved by the University of Miami Institutional Review Board (IRB) (Protocols 2015-1022, 2018-0822, 2019-0756) and the National Health Research Ethics Committee of Nigeria (NHREC/01/01/2007- 23/08/2019).

### Study Population

All women diagnosed with ovarian cancer between 2005 and 2017 (in Florida) and July 2016-July 2019 (in Nigeria) were identified through the state cancer registry (Florida) or each institution’s Department of Pathology (Nigeria), respectively. Only data for cases with self-identification as non-Hispanic White, non-Hispanic Black, referred to as Caribbean-born Black or US-born Black in this report, were included.

Categories for histologic type included: EOC (serous, clear cell, endometrioid, mucinous and carcinosarcoma); germ cell tumors (immature teratoma, dysgerminoma, endodermal sinus tumor [yolk sac tumor], choriocarcinoma and carcinoid); SCST (granulosa cell and Sertoli-Leydig cell tumors). Histology and grade were reviewed by consortia lead Pathologists (AP and AO) to ensure consistency, with tumors being segregated into low-grade (low/moderately differentiated) or high-grade (poorly differentiated), when applicable. Borderline ovarian cancer histotypes were not included. If the grade and histology were inconsistent (e.g. carcinosarcoma classified as low-grade), they were adjusted through pathologist review to meet WHO classifications of the ovary.

In Nigeria, data extracted from pathology reports were captured in REDCap, a mature, secure web application which is encrypted, HIPAA-compliant, and hosted on a server at the University of Miami. Variables obtained included date of diagnosis, age of diagnosis in years, treatment facility (by country and state, if relevant), tumor grade and histology when available. Age was recorded as a continuous variable. Patients’ country of birth was classified as US-born, Caribbean-born, or West African-born. Country of birth, race, and ethnicity were all self-reported.

### Consortium

The Transatlantic Gynecologic Cancer Research Consortium (TAGCReC) was established to facilitate gynecologic cancer research across the nations that represent African diaspora people. TAGCReC’s primary mission is to take a comprehensive approach to gynecologic oncology challenges across this population and to leverage opportunities present at our different institutions. The consortium’s focus areas include cancer prevention, treatment, and survivorship, with transnational education opportunities. We have used this platform to interrogate race, genetics, environment, and health care practices to address health and health disparities in Africa and across the diaspora. Currently, the consortium is comprised of members in Nigeria, the USA, and the Caribbean; members include gynecologists with an interest in gynecologic oncology, board certified gynecologic oncologists, general and gynecologic pathologists, molecular geneticists, epidemiologists, and behavioral and basic scientists. The members were linked initially through existing African diasporic consortia and societies in Nigeria (Gynecological Oncology Society of Nigeria), the Caribbean (Caribbean Gynecologic Cancer Society), and USA (African Cancer Consortium – AC3).

### Data Sources

Data from the Surveillance, Epidemiology and End Results (SEER) (13) were used to capture nationwide ovarian data cancer data for comparison. Variables obtained from SEER included race/ethnicity, age ranges, tumor characteristics such as stage at diagnosis, histology, and grade. Country of birth is not an accessible variable. The age range categories provided in SEER were used to compare Nigerians, Black and White populations in the US by cancer histotype.

Florida Cancer Data System (FCDS) is the legislatively mandated, population-based central cancer registry for Florida. Cases are abstracted from patient medical records in hospitals, free-standing ambulatory surgical facilities, radiation therapy facilities, private physicians, and death certificates codes (14, 15). Variables obtained from FCDS included age, race/ethnicity, patients’ country of birth, and tumor characteristics such as stage at diagnosis, histology, and grade. Race/ethnicity was based on self-identification and was present in nearly all (more than 98%) of the health records. These data are de-identified and therefore exempt from IRB approval. Women were included if they self-identified as Black and were born in the United States or one of the English- or French speaking Caribbean nations (Anguilla, Antigua, Bahamas, Barbados, Belize, British Virgin Islands, Cayman Islands, Cuba, Dominica, British Guyana, Grenada, Haiti, Jamaica, St. Kitts and Nevis, St. Lucia, St. Vincent, Trinidad Tobago, Turks and Caicos Islands, Suriname, US Virgin Islands or West Indies). Women who were born in Africa were excluded from the analysis due to low representation (1 Algeria, 1 Kenya, 1 Morocco, 2 Nigeria, 1 Tanzania, 2 Uganda and 1 South Africa).

The ovarian cancer cases were identified using International Classification of Diseases for Oncology, *Tenth Revision* (ICD-10). Tumor site and histology codes included in the analysis were as follows: primary site (C56.9, C57.0) classified as malignant tumors; serous (8050, 8120, 8122, 8130, 8140, 8201, 8260, 8440–8442, 8450, 8452, 8460–8463, 9014); clear cell (8005, 8310, 8313, 8443, 8444); endometrioid (8290, 8380–8383); carcinosarcoma (8575, 8950, 8951, 8980, 8981); mucinous (8144, 8384, 8470–8472, 8480–8482, 9015); mixed, undifferentiated, unspecified carcinoma (other/NOS; 8000–8004, 8010, 8020–8022, 8030–8033, 8046, 8052, 8070–8072, 8074, 8084, 8230, 8255, 8261–8263, 8323, 8560, 8562, 8570, 8574, 8940, 9000) (16). Non-epithelial histologic types; GCT - 9060, 9070, 9071, 9082, 9100, and SCST – 8620-8623, 8630-8633) were also included. Similarly to SEER, we used FCDS data to compare age range categories in Nigerians, Caribbean born blacks, US born blacks and US born whites. We also used age numerical values to create boxplots comparing these four groups. Finally, the percentages of tumor histotypes across four groups were compared: Epithelial ovarian cancer types between Nigerians, CB Blacks and USB Blacks (Serous vs Non-serous).

## Globocan

GLOBOCAN 2020 provides cancer incidence estimates across 185 countries or territories by sex and age. For ovarian cancer, incidence and mortality data were available for World Health Organization (WHO) regions as well as individual countries and territories. We selected ovarian cancer incidence and mortality data for Africa, Latin America, and the Caribbean, and extracted age-standardized rates; data on cancer histotype were not available (17).

### Statistics

Statistical analyses were performed using STATA IC 14.2 (StataCorp, College Station, TX); SAS software 9.4 (SAS Institute Inc., Cary, NC) and Prism 9.1.2 (Graphpad Prism LLC). The R packages ggpubr and ggplot2 (16) were used to create line charts and boxplots showing the age at cancer diagnosis distribution across subgroups. Summary statistics were used to describe the patient cohorts. Wilcoxon rank-sum was used for continuous variables in nonparametric distributions. Associations between categorical covariates and continuous variables were assessed with chi-squared tests and independent sample *t*-tests, respectively. Differences across means in all subgroups were tested using the analysis of variance (ANOVA). All tests were 2-tailed and p-value of < 0.05 was statistically significant.

## Results

**Figure 1:**
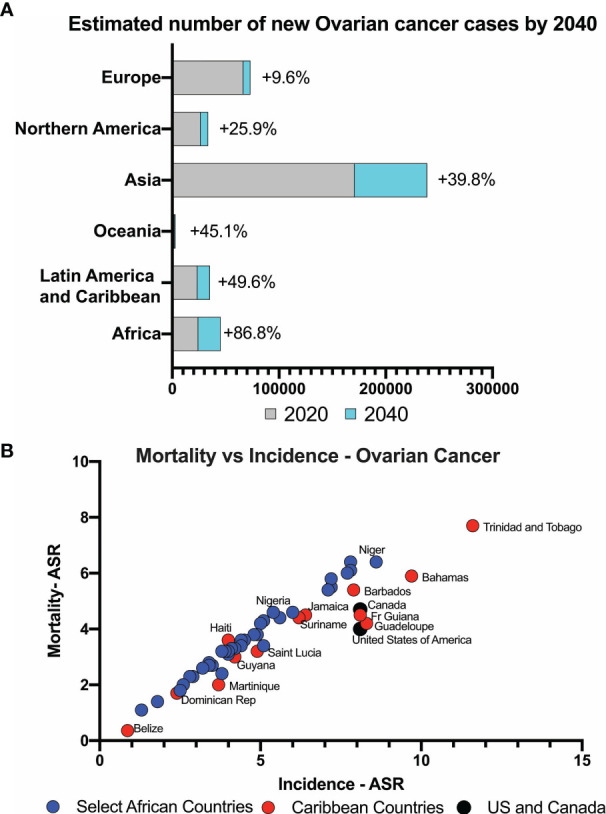
**(A)** Estimated number of ovarian cancer cases expected globally. Low- and middle-income countries expect to see significant increase in newly diagnosed cases. **(B)** Mortality versus incidence rates of ovarian cancer in countries with majority Black women compared to USA and Canada.

Interrogation of the WHO GLOBOCAN 2020 data showed that globally, 313 959 incident ovarian cancer cases were forecasted in 2020. These numbers are expected to increase by 86.8% in Africa and 49.6% in Latin America and the Caribbean by 2040, compared to 25.9% in North America and 9.6% in Europe (**Figure 1A**). In Africa, ovarian cancer has the 5^th^ highest incidence rate among cancers in women (3.8%) (ASR, 5.4/100,000), whereas in the Caribbean (26 countries) and the US, new ovarian cancer cases rank 9^th^ (4.6/100,000) and 11^th^ (8.0/100,000) respectively. The mortality to incidence ratio is high both in African and Caribbean countries when compared to North America countries such as the United States and Canada. In Africa, the age-standardized incidence rate (ASR) is highest in Ghana at 8.6/100,000, 5.6/100,000 in Nigeria and lowest in Mozambique at 1.8/100,000. In the Caribbean, the ASR was highest in Trinidad and Tobago (11.6/100,000) and lowest in Belize (0.87/100.000). The ratio of mortality to incidence in West and East African countries is high at 0.97, similar to the Caribbean ratio of 0.92 (**Figure 1B**).

**Figure 2:**
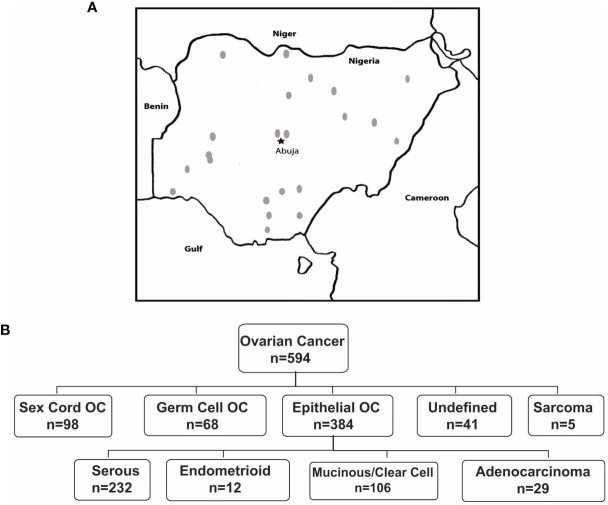
**(A)** Study sites across Nigeria that participated in study. **(B)** Distribution of ovarian cancer cases by histology in Nigeria.

**Figure 3:**
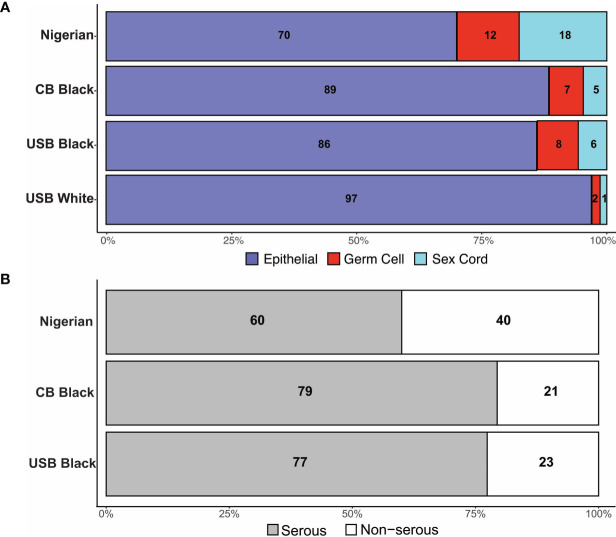
**(A)** Distribution of tumor histology by cohort. **(B)** Distribution of Serous tumor types in Black women.

In Nigeria, data were collected in two phases. During Phase I, members were asked to identify within their institutions all ovarian cancer cases diagnosed within a 12-month period (June 2018-July 2019), with reported age and histotype. Twenty-one (21) sites across 6 political geographic regions in Nigeria provided data. In Phase II, in which 16 sites (12 from Phase I and 4 additional sites) participated, an independent pathology review of available archival tissue from ovarian cancer cases diagnosed between 2017-2019 was performed (**Supplementary Table 1** and **Figure 2A**). In total, 621 cases were identified. Of these, 594 cases had a confirmatory pathology report and/or tissue blocks. Epithelial tumors were the most common (**Figure 2B**), comprising 64.7% of cases, followed by SCST (16.2%), GCT (11.5%), sarcoma (0.8%) and other (lymphoma, Brenner and undefined tumors, 6.9%)). Epithelial tumors were serous (n=232, 60.4%); mucinous (n=97, 25.2%); endometrioid (n=12, 3.1%); clear cell (n=9,2.3%); carcinosarcoma (n=5, 1.3%) and undefined (7.6%). There were differences in the proportions of tumor types reported by regions across Nigeria (**Supplementary Table 1**). Nigerians had a higher proportion of GCT relative to the other groups assessed from the African diaspora (**Figure 3A**). Of the epithelial ovarian cancer (EOC) cases, Nigerians (60.4%) had the smallest proportion of serous cancers, compared to Caribbean-born Blacks (CBB, 79.4%) and US-born Blacks (USB, 77.4%) cases, p<0.0001 (**Figure 3B** and **Supplementary Table 2**).

**Figure 4:**
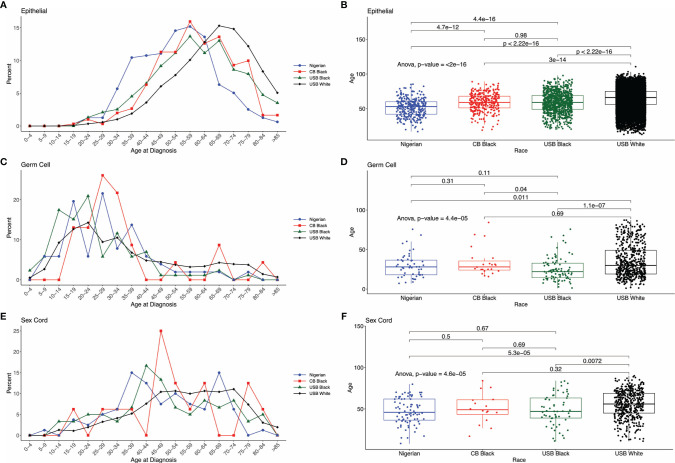
Distribution of cases by age across the comparative groups. **(A, C, E)** Histograms by histologic type EOC, Germ Cell and Sex Cord Stromal tumors. **(B, D, F)** ANOVA comparing mean age at cancer diagnosis across different cohorts and histologic type.

Age at diagnosis for the three major histologic types – epithelial, sex cord stromal, and germ cell – varied significantly across Nigerian, CBB, USB and US White women (**Table 1** and **Figures 4A**–**F**). In our comparative analysis of the FCDS and Nigerian ovarian cohorts, the mean age of EOC patients in Nigeria was significantly younger, 51.7± 12.8 years (95% CI 17-80), than USB (58.9 years, 95% CI 21-88) and CBB (59.0 years, 95% CI 23-87) (p<0.001). SEER data showed a similar trend where Nigerians diagnosed with EOCs skewed to the left of US Black and White EOC patients (**Supplementary Figure 1**). Whereas there were non-significant differences amongst the ethnic minorities, Black women, independent of country of birth (West Africa, USA, or the Caribbean), were diagnosed at a younger age with both germ cell (p=0.011) and SCST compared to White women (p<0.01) (**Table 1** and **Figure 4C**–**E**). We assessed stage at presentation of incident ovarian cancer cases in Florida. The rare histologic tumors, germ cells and sex cord stromal tumors, there were no significant difference at stage at presentation. However, with epithelial ovarian cancers, there was a significant difference in stage at diagnosis amongst the three groups: CBB, USB and majority White EOC population. Both CBB and USB EOC patients had higher proportions of stage 3-4 and unstaged diagnoses (p=0.0006, **Supplementary Table 3**).

**Table 1:** Distribution by age at diagnosis and ovarian histotype across groups.

	Number of Cases	Mean Age (± SD)	Median	p-value^a^
**Epithelial Ovarian Tumor**				Overall <0.001
Nigerian	316	51.7 ± 12.8	53	Ref
CBB	301	59.0 ± 13.0	59	<0.001
USB	906	58.9 ± 15.0	59	<0.001
US White NH	33377	65 ± 13.4	66	<0.001
**Germ Cell Tumor**				Overall <0.001
Nigerian	51	29.5 ± 15.1	28	Ref
CBB	23	33.9 ± 17.9	28	0.31
USB	86	25.2 ± 15.0	22	0.11
US White NH	560	35.4 ± 20.5	30	0.011
**Sex Cord Stromal Tumor**				Overall < 0.001
Nigerian	80	47.5 ± 15.9	46	Ref
CBB	16	50.9 ± 18.3	49	0.5
USB	60	48.8 ± 18.3	47	0.67
US White NH	461	55.6 ± 16.5	56	<0.011

a ANOVA. CBB, Caribbean-born Black; USB, US-born Black; NH, non-Hispanic.

## Discussion

In 2020, ovarian cancer will account for about 313,000 cases worldwide, representing 3.4% of global cancer incidence in women, but 4.7% of cancer-related deaths. Our analysis of GLOBOCAN data revealed that the burden of ovarian cancer is increasing annually. By 2040, there is an expected 49.6-86.8% increase in ovarian cancer incidence in Africa, Latin America, and the Caribbean, which is much greater than the modest 9.6-25.9% increase expected among the predominantly White regions of Europe and North America.

Black women in Africa and the African diaspora develop distinct proportions of ovarian cancer histotypes across ancestry, ethnicity, and geography. The geographic regions in this study represent both indigenous Africans and diasporic Africans through forced immigration and enslavement, and now including voluntary emigration. As a result of this movement of people, African diaspora populations are genetically admixed (12, 18, 19). In the US, substantial demographic data collected on cancer patients demonstrate health disparities among people of African ancestry. Black women in the US have lower incidence of ovarian cancer but continue to experience worse outcomes. Smaller studies in West African countries such as Nigeria have documented similar poor survival in women diagnosed with ovarian cancer (20, 21).

Known factors that both modify and predict development of ovarian cancer include family history of breast and/or ovarian cancer; fertility factors such as decreased parity, earlier onset of menses, smaller family size, later age at time of first pregnancy, reproductive behavior; and environment. Risk factors associated with race and ethnicity are less well defined. Among populations with West or East African ancestry, these risk factors are poorly understood or in some cases not known at all. Our data collected in Nigeria represent a large-scale collection from the 7^th^ most populated country in the world with a population of 206 million people, in which there are over 250 ethnic groups.

Our data show that there was a significant difference in the proportions of serous cancer across the three international cohorts. In addition, Black women diagnosed with EOC are diagnosed at more advanced stages, as have been shown in larger SEER data (11, 22). Nigerian women had a lower proportion of EOC cases, an observation previously published in a single institutional study (23–25). Further, differences in reported ovarian histotypes across Nigeria potentially suggest different cultural – health behavioral practices and thus exposures that may influence risk of ovarian cancer histologic development. Additionally, there may be unknown genetic, reproductive, and biologic influences (e.g. unreported prevalence of endometriosis, a precursor to clear cell and endometrioid ovarian cancers) as contributors to ovarian cancer histotypes. In the US, sex cord stromal tumors (SCST) only represent approximately 1% of ovarian cancer cases in White women, but a higher percentage in USB (6%) and CBB (5%) women; Nigerian women have the highest percentage (18%).

In Nigeria, 1 in 8 women diagnosed with breast cancer has hereditary breast and ovarian cancer syndrome (HBOC) (26). Women with HBOC develop cancers at younger ages that are typically high-grade, and of serous histology. Among CBB, the percentage of women with breast or ovarian cancer who have HBOC is variable: the Bahamas, 24%, with a pathogenic variant in *BRCA1, BRCA2* or *RAD51C*; Trinidad and Tobago, 12%; and Jamaica, 3% (27–30). No such data about ovarian cancer risk specifically in women in Africa are currently available; at present it is unknown if the histologic prevalence described here is representative of variations in HBOC or other hereditary cancer syndromes.

Reproductive factors like gravidity, parity and age at pregnancies influence ovarian cancer risks and etiology. A Norwegian study reported that pregnancy and age at the first and last births are sometimes associated with an differential risks of developing non-EOC malignancies (31). More specifically for SCST a decrease in risk was observed with increasing age at last birth. In contrast, increasing age at first and last births was associated with an increased risk for GCT. The total fertility rate (TFR, the average number of children per woman) in Nigeria is 5.3. This ranged from 3.9 in the southwest to 6.6 in the northwest. The lowest rate was Lagos state with 3.4, and the highest was Katsina state with 7.3. On average in Nigeria, women aged 45-49 years have given birth to 6.4 children, with 2% never having given birth at all (infertile rather than voluntarily childless) (32). Total fertility rates in the Caribbean range from 2.96 in Haiti to 1.44 in St. Lucia, a steady decline from the 1950s when the TFR was 5.4 for the entire region (33). Similarly, in the USA, Black women have experienced a decrease in TFR to 1.8, compared to 1.7 in US White women (34). These differences in TFRs may suggest fertility correlations with non-EOC tumors, although more data are required to substantiate observations about higher incidence of these rare ovarian tumors in all Black women.

Women in Nigeria were also diagnosed at a younger age with EOC compared to Black women in the US and the Caribbean. There are reproductive factors such as age at first pregnancy and number of pregnancies that may account for this observation. Additionally, broadly in Western Africa life expectancy is 61 years compared to 75 years in the Caribbean and 83 years in the USA. Early age at menarche, multiparous and shorter lifespan, as opposed to living longer and having the opportunity to age into developing higher-risk disease, may explain, in part, the 10-year shift in EOC age at diagnosis among Nigerian women, CBB, and USB. A recent report from northern Nigeria (Zaria) reported that 80% of patients with ovarian cancer were pre-menopausal (24). Environmental factors such as air quality, and social determinants of health including housing, access to healthy food, equitable and proper health infrastructure, and systemic racism (in the US) are variables that can influence ovarian cancer etiology across the three regions studied. These variables are known to modulate genetic expression through epigenetics and will be important to assess disease pathogenesis in future investigations (35). The differences in the proportion of ovarian cancer histotypes between USB, CBB, and Nigerians undoubtedly involve a complex underpinning of genetic, epigenetic, lifestyle, and reproductive factors, which must be explored.

### Limitations

As a pilot study, this work includes data from only one West African country. The findings may not necessarily be applicable to other geographic locations and should be considered hypothesis-generating. There is a selection bias in that the US-based cohort was drawn from Florida, which may not be representative of other North American regions. Further the cohort in Nigeria will only capture women who had resources (financial and social) to attend the clinics in participating sites. Comprehensive clinicopathologic data on each case were not available for all patients during the pilot study interval. There is limited chronological overlap between the US and Nigerian cohorts which may influence incident cases between the regions. A more thorough medical chart review, currently ongoing, is expected to reveal additional reproductive, familial, and epidemiologic factors associated with different types of ovarian cancers observed in these populations, and perhaps can better explain our preliminary observations.

## Conclusion

Ovarian cancer in some West African and Caribbean countries is highly prevalent. The differences in prevalence and type are more pronounced in West African ovarian cancer cases for which genetics (familial), environmental, and reproductive factors may influence the etiology and pathogenesis in the spectra of ovarian cancer histotypes observed. Further, the exponential increase in ovarian cases expected by 2040 in Africa and the Caribbean, regions with low- to middle-income countries, highlights the need not only for robust health system infrastructure to decrease the mortality burden, for comprehensive studies to better understand the complex etiologies of this disease. Currently, there are no screening guidelines for ovarian cancer beyond risk-reduction surgeries and opportunistic salpingo-oophorectomies. It will be important to study both epidemiology and genetic factors influencing ovarian cancer pathogenesis in the diverse group of women across the African diaspora to identify factors contributing to both higher incidence and mortality. Such research will ultimately inform strategies for cancer prevention, early detection, and treatment optimization for these routinely underserved populations.

## Data Availability Statement

The original contributions presented in the study are included in the article/**Supplementary Material**. Further inquiries can be directed to the corresponding authors.

## Ethics Statement

The studies involving human participants were reviewed and approved by University of Miami and the National Health Research Ethics Committee of Nigeria. Written informed consent for participation was not required for this study in accordance with the national legislation and the institutional requirements.

## Author Contributions

Design and Conduct of the Study: SG, BA, MS, AP, and AO. Provision of study materials or patients: All authors. Collection and assembly of data: SG, AO, AP, MC, AM, MS, BA. Data analysis and interpretation: SG, AO, AP, BA, MS, AM, AS-C. Preparation and review Manuscript: All authors. Accountable for all aspects of the work: All authors. All authors contributed to the article and approved the submitted version.

## Funding

The study was partially funded by the HERI Foundation (BA, SG, MS, AP) and Sylvester Comprehensive Cancer Center. Research reported in this publication was supported by the National Cancer Institute of the National Institutes of Health under Award Number P30CA240139. The funders had no specific role in design and conduct of the study; collection, management, analysis, and interpretation of the data; preparation, review, or approval of the manuscript; and decision to submit the manuscript for publication.

## Conflict of Interest

The authors declare that the research was conducted in the absence of any commercial or financial relationships that could be construed as a potential conflict of interest.

## Publisher’s Note

All claims expressed in this article are solely those of the authors and do not necessarily represent those of their affiliated organizations, or those of the publisher, the editors and the reviewers. Any product that may be evaluated in this article, or claim that may be made by its manufacturer, is not guaranteed or endorsed by the publisher.

## Supplementary Material

Supplementary Figure 1 Distribution of cases by age across comparative groups using SEER data. Black, White, and Nigerian cohorts. **(A)**. Nigerian women diagnosed with EOC skew left of both Black and White women using SEER categorical age distribution. **(B)**. No observed differences in age at diagnosis between Nigerian women Germ Cell versus women in the US. **(C)**. No observed differences in age at diagnosis between Nigerian women Sex Cord Stromal tumors versus women in the US.
