# Supplementary figures and images for: An Assessment of Ovarian Cancer Histotypes Across the African Diaspora
Source: Front Oncol. 2021 Nov 26;11:732443. doi: 10.3389/fonc.2021.732443 (PMC8662547; doi:10.3389/fonc.2021.732443)

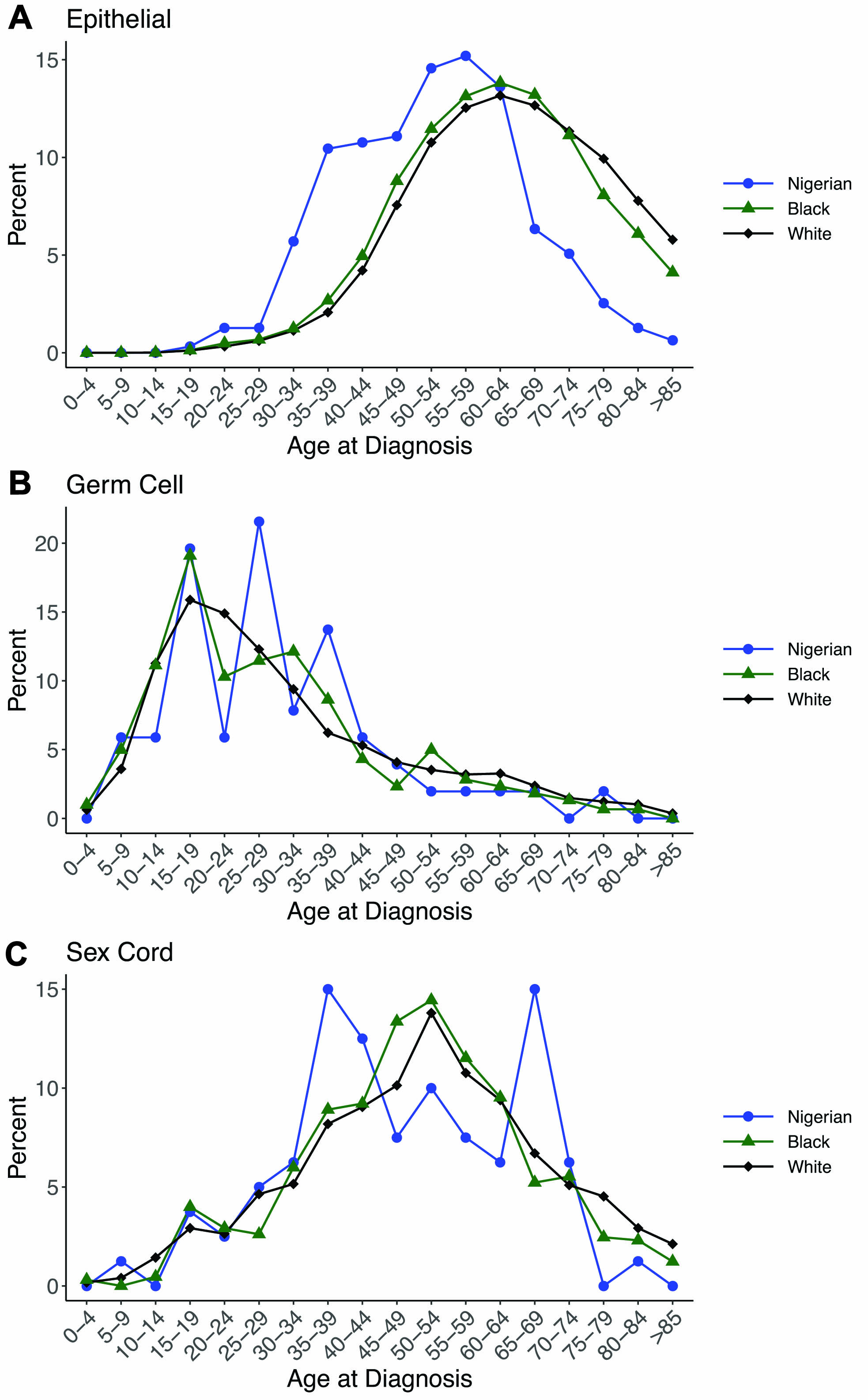

Supplement: Supplementary Figure 1 — Distribution of cases by age across comparative groups using SEER data. Black, White, and Nigerian cohorts. (A). Nigerian women diagnosed with EOC skew left of both Black and White women using SEER categorical age distribution. (B). No observed differences in age at diagnosis between Nigerian women Germ Cell versus women in the US. (C). No observed differences in age at diagnosis between Nigerian women Sex Cord Stromal tumors versus women in the US. [file Image_1.jpeg]
