# Supplementary material for: An Assessment of Ovarian Cancer Histotypes Across the African Diaspora
Source: Front Oncol. 2021 Nov 26;11:732443. doi: 10.3389/fonc.2021.732443 (PMC8662547; doi:10.3389/fonc.2021.732443)
Supplement: Supplementary file 2 [file Table_1.docx]

**Supplementary Table 1. Distribution of cases by Study site and Tumor type in Nigeria**

| Site | Number of Cases Total n=594 (%) | Tumor Subtype | | | |
| --- | --- | --- | --- | --- | --- |
|  |  | Epithelial | Germ Cell | Sex Cord Stromal | Sarcoma/  Other |
| Amuinu Kano Teaching Hospital, Kano | 69 (11.6) | 48 | 13 | 5 | 3 |
| UCH Ibadan University College Hospital, Ibadan | 60 (10.1) | 45 | 6 | 9 | 0 |
| University of Nigeria Teach Hospital, Enugu | 46 (7.7) | 27 | 5 | 13 | 1 |
| Federal Medical Center, Yola | 45 (7.6) | 19 | 8 | 6 | 2 |
| Federal Medical Center/General Hospital, Katsina | 42 (7.1) | 33 | 4 | 3 | 2 |
| University of Abuja Teaching Hospital, Gwagwalada | 35 (5.9) | 22 | 2 | 6 | 5 |
| Nnamdi Azikiwe University Teaching Hospital, Nnewi | 34 (5.7) | 22 | 3 | 8 | 1 |
| Federal Teaching Hospital, Gombe | 32 (5.4) | 11 | 6 | 11 | 4 |
| Federal Medical Centre, Owerri | 28 (4.7) | 12 | 1 | 2 | 13 |
| Abubakar Tafawa Balewa University Teaching Hospital, Bauchi | 28 (4.7) | 20 | 2 | 3 | 3 |
| Ahmadu Bello University Teaching Hospital, Zaria | 21 (3.5) | 15 | 1 | 5 | 0 |
| University Of Calabar Teaching Hospital, Calabar | 20 (3.4) | 16 | 2 | 2 | 0 |
| Alex Ekwueme Federal Teaching Hospital, Abakaliki | 19 (3.2) | 7 | 4 | 6 | 2 |
| Lagos University Teaching Hospital, Lagos | 18 (3.0) | 16 | 0 | 1 | 1 |
| National Hospital, Abuja | 17 (2.9) | 8 | 2 | 3 | 4 |
| Obafemi Awolowo University Teaching Hospitals Complex, Ile-Ife | 15 (2.5) | 11 | 3 | 0 | 1 |
| University Of Maiduguri Teaching Hospital, Maiduguri | 14 (2.4) | 9 | 1 | 4 | 0 |
| Usmanu Danfodiyo University Teaching Hospital Sokoto, Sokoto | 14 (2.4) | 4 | 3 | 6 | 1 |
| University of Ilorin Teaching Hospital, Ilorin | 9 (1.5) | 7 | 0 | 2 | 0 |
| University of UYO Teaching Hospital, Uyo | 8 (1.3) | 7 | 0 | 0 | 1 |
| University of Port Harcourt Teaching Hospital | 7 (1.2) | 5 | 1 | 1 | 0 |
| Federal Medical Center, Birnin Kebbi | 7 (1.2) | 5 | 1 | 1 | 0 |
| Federal Medical Center, Birnin Kudu | 3 (0.5) | 2 | 0 | 1 | 0 |
| LAUTECH Teaching Hospital, Osogbo | 3 (0.5) | 3 | 0 | 0 | 0 |
