# Supplementary material for: An Assessment of Ovarian Cancer Histotypes Across the African Diaspora
Source: Front Oncol. 2021 Nov 26;11:732443. doi: 10.3389/fonc.2021.732443 (PMC8662547; doi:10.3389/fonc.2021.732443)
Supplement: Supplementary file 3 [file Table_2.docx]

**Supplementary Table 2. Distribution of serous vs non-serous by nativity**

| **Proportion** | Serous | Non-serous | p-value |
| --- | --- | --- | --- |
| US-born | 701 (77.4%) | 205 (22.6%) | <0.0001^†^ |
| Caribbean-born | 239 (79.4%) | 62 (20.6%) |  |
| Nigeria | 232 (60.4%) | 152 (39.6%) |  |
|  |  |  |  |
| **Age** (mean (SD)) | Serous | Non-serous |  |
| US-born | 59.6 (14.9) | 56.8 yrs (14.7) | 0.0175^‡^ |
| Caribbean-born | 60.4 (12.2) | 53.5 yrs (14.6) | <0.0001^‡^ |
| Nigeria | 51.9 (12.3) | 51.3 yrs (13.6) | 0.70 ^b^ |

^†^Chi-squared, ^‡^Student’s t-test.
