# Supplementary material for: An Assessment of Ovarian Cancer Histotypes Across the African Diaspora
Source: Front Oncol. 2021 Nov 26;11:732443. doi: 10.3389/fonc.2021.732443 (PMC8662547; doi:10.3389/fonc.2021.732443)
Supplement: Supplementary file 4 [file Table_3.docx]

**Supplementary Table 3. Stage of cancer at diagnosis by place of birth**

*Categories excluded from the analysis.

^†^Chi-square comparing all stages.

^‡^Chi-square comparing stage 1-2 vs stage 3-4.

| STAGE | CBB  N (%) | USB  N (%) | WNH  N (%) | p-value |
| --- | --- | --- | --- | --- |
| **Epithelial Ovarian Cancer**^†^ |  |  |  | 0.0006 |
| Stage 0 (In Situ)* | 1 (0.5) | 0 | 26 (0.2) |  |
| Stage 1 | 16 (7.5) | 53 (11) | 1776 (12.4) |  |
| Stage 2 | 23 (11) | 64 (13.4) | 2154 (15) |  |
| Stage 3 | 5 (2.5) | 7 (1.5) | 145 (1) |  |
| Stage 4 | 146 (69.5) | 332 (68.9) | 9661 (67.4) |  |
| Unstaged | 19 (9) | 25 (5.2) | 565 (4) |  |
| TOTAL | 210 | 481 | 14327 |  |
| **Germ Cell**^‡^ |  |  |  | 0.8597 |
| Stage 0 (In Situ)* | 0 | 0 | 0 |  |
| Stage 1 | 8 (53.3) | 27 (62.8) | 125 (48.4) |  |
| Stage 2 | 2 (13.3) | 5 (11.6) | 51 (19.8) |  |
| Stage 3 | 0 (0) | 2 (4.7) | 6 (2.3) |  |
| Stage 4 | 3 (20) | 9 (20.9) | 64 (24.8) |  |
| Unstaged* | 2 (13.4) | 0 (0) | 12 (4.7) |  |
| TOTAL | 15 | 43 | 258 |  |
| **Sex Cord Stromal**^‡^ |  |  |  | 0.1417 |
| Stage 0 (In Situ)* | 0 | 0 | 0 |  |
| Stage 1 | 8 (61.6) | 14 (41.2) | 113 (44.8) |  |
| Stage 2 | 3 (23) | 10 (29.4) | 47 (18.7) |  |
| Stage 3 | 0 | 0 | 2 (0.8) |  |
| Stage 4 | 2 (15.4) | 6 (17.6) | 79 (31.4) |  |
| Unstaged* | 0 | 4 (11.8) | 11 (4.3) |  |
| TOTAL | 13 | 34 | 252 |  |
